# Feasibility of Manufacturing Strand-Based Wood Composite Treated with β-Cyclodextrin–Boric Acid for Fungal Decay Resistance

**DOI:** 10.3390/polym12020274

**Published:** 2020-01-29

**Authors:** Lili Cai, Hyungsuk Lim, Nicholas C. Fitzkee, Bojan Cosovic, Dragica Jeremic

**Affiliations:** 1 Department of Forest, Rangeland and Fire Sciences, University of Idaho, Moscow, ID 83844, USA; lcai@uidaho.edu; 2 Department of Sustainable Bioproducts, Mississippi State University, Starkville, MS 39759, USA; bc1857@student.exchange.msstate.edu; 3 Department of Chemistry, Mississippi State University, Starkville, MS 39762, USA; NFitzkee@chemistry.msstate.edu

**Keywords:** cyclodextrin, boric acid, wood preservatives, fungi-resistant, internal bond, wood composites

## Abstract

The feasibility of using β-cyclodextrin (βCD) as an eco-friendly carrier of boric acid for the protection of strand-based wood composites against decay fungi was evaluated. The formation of a βCD–boric acid (βCD–B) complex was confirmed by the appearance of the boron–oxygen bond by using attenuated total reflection–Fourier transform infrared spectroscopy. Chemical shifts of around 6.25 and 1.41 ppm were also observed in ^1^H Nuclear Magnetic Resonance (NMR) and ^11^B NMR spectra, respectively. The βCD–B preservatives at two levels (5 and 10 wt.%) were uniformly blended with southern pine strands that were subsequently sprayed with polymeric methylene diphenyl diisocyanate (pMDI) resin. The blended strands were formed into a loose mat by hand and consolidated into 25 × 254 × 12 mm oriented strand boards (OSB) using a hot-press. The OSB panels were cut to end-matched internal bonding (IB) strength and fungal decay resistance test specimens. The vertical density profiles (VDPs) of the IB specimens were measured using an X-ray based density profiler and the specimens with statistically similar VDPs were selected for the IB and decay tests. The IB strength of the treated specimens was lower than the control specimens but they were above the required IB strength of heavy-duty load-bearing boards for use in humid conditions, specified in the BS EN 300:2006 standard. The reduced IB of preservative-treated OSB boards could be explained by the destabilized resin upon the addition of the βCD–B complex, as indicated by the differential scanning calorimetry (DSC) results. The resistance of the OSB panels against two brown-rot fungi (i.e., *G. trabeum* or *P. placenta*) was evaluated before and after accelerated leaching cycles. The treated OSBs exposed to the fungi showed an average mass loss of lower than 3% before leaching, while the untreated OSBs had 49 and 35% mass losses due to decay by *G. trabeum* or *P. placenta*, respectively. However, upon the leaching, the treatment provided protection only against *G. trabeum* to a certain degree (average mass loss of 15%). The experimental results suggest that protection efficacy against decay fungi after leaching, as well as the adhesion of the OSB strands, can be improved by increasing the amount of pMDI resin.

## 1. Introduction

The protection of wood composites by various biocides has been practiced since the end of the last century [1]. Most of the conventionally used preservatives are either heavy metal based compounds (e.g., chromated copper arsenate) or synthetic compounds (e.g., pentachlorophenol), which are considered toxic and harmful to human beings and our environment [2,3]. As one of the oldest preservatives, boron exhibits a low level of toxicity to mammals and the environment [4]. Borate-treated wood products are not corrosive to metal fasteners and are colorless and odorless after treatment [5]. Additionally, borates can easily penetrate into the wood at a higher moisture content, making them ideal for treating refractory wood species, such as Douglas fir [6]. However, due to the mobility of borates in aqueous solutions, borates are vulnerable to leaching, which considerably reduces the life-span of borate-treated wood products in outdoor applications.

To reduce the leaching of borates from wood composites, various methods have been adopted [4,7,8]. One of the most common approaches is treating wood composites with trimethyl borate [9], a type of borate ester that can vaporize under high-temperature conditions. In the vapor phase, this chemical will penetrate into the panels and react with residual water. The deposited boric acid in the vapor-treated composites will ultimately leach out, due to the inherent solubility of boric acid in water. An alternative approach is the use of less water-soluble boron-based compounds, such as zinc borates, and blending the preservatives with resin, wax and wood furnish. This method currently dominates the in-process treatment of wood composites in the U.S. and Canada [10]. However, zinc borates significantly reduce the internal bonding strength of the panels [11]. Thus, it is imperative to seek alternative boron-based preservatives with an improved leaching performance, which are also compatible with common resin systems.

β-Cyclodextrin (βCD) is a cyclic oligosaccharide formed naturally by the action of the glucosyltransferase enzyme on starch [12]. It consists of 7 D-glucopyranoside units linked by α−1, 4 glycosidic bonds in a ring. This unique structure provides βCD with a hydrophilic exterior and hydrophobic interior, along with multiple hydroxyl groups. This amphiphilic molecule has been used as a carrier to partially immobilize boric acid through the formation of a covalent bond [13]. The resultant βCD–boric acid complex (βCD–B) has high thermal stability and decomposes at temperatures above 200 °C [14], making it an attractive treatment for wood composite wafers prior to hot pressing. In our previous study, we have demonstrated the use of βCDs as sustained released carriers for natural volatile compounds (i.e., mustard oil and essential oils) for the protection of wood and wood composites [15,16,17]. Besides acting as a carrier and immobilizer of boric acid, βCDs can also crosslink with polymeric methyl diphenyl di-isocyanate resin (pMDI) [18,19], which could further improve the mechanical properties of boards treated with boron-based preservatives. To our knowledge, the effect of βCD–B on the long-term durability of the wood composites has not been evaluated, and this study thus examines the feasibility of βCD–B for oriented strand board (OSB) manufacturing. The preservative treatment will be especially beneficial for OSB panels subjected to any moisture exposure, which increases biodeterioration risk, during their use. βCD was used to partially immobilize boric acid, and the powder complex was applied during the OSB manufacturing process. The leaching, fungicidal, and cohesion properties of the boards were evaluated.

## 2. Materials and Methods

### 2.1. Preparation of βCD–B Complex

**Figure 1:**
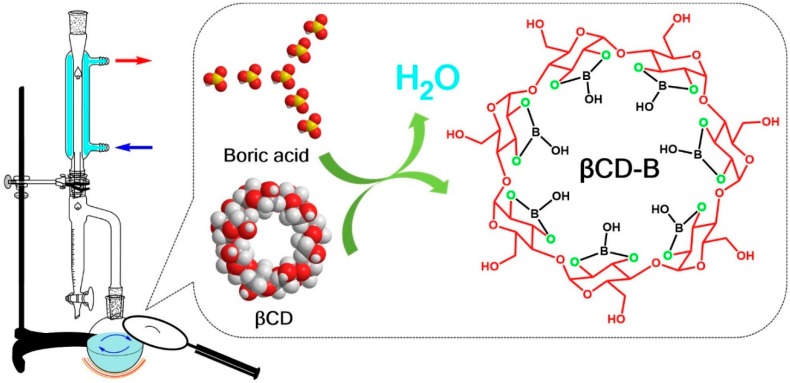
Schematic process for preparing β–cyclodextrin-boric acid (βCD–B) complex.

The βCD–B complex was prepared by mixing βCD (Acros Organics, New Jersey, USA) with boric acid (Fisher Chemical, New Jersey, US) at a molar ratio of 1:7 and refluxing the mixture in toluene (Fisher Chemical, New Jersey, USA) in a Dean–Stark apparatus for 18 h [13] (Figure 1). The residual toluene was removed in a rotary evaporator and the resultant mixture was kept in a desiccator prior to analysis. The physical mixture of βCD and boric acid was obtained by homogenizing the two powders using the same molar ratio.

### 2.2. Confirmation of βCD–B Complex

#### 2.2.1. Attenuated Total Reflection–Fourier Transform Infrared Spectroscopy (ATR-FTIR)

The formation of the βCD–B complex was confirmed by a PerkinElmer Spectrum Two FTIR spectrometer equipped with a universal ATR (PerkinElmer, Waltham, MA, USA). Three replicates of ATR–FTIR spectra were obtained for every sample in the spectral range of 4000 to 400 cm^−1^ at a resolution of 4 cm^−1^. All the spectra were baseline-corrected interactively using Spectrum^®^ Quant software (PerkinElmer, Waltham, MA, USA).

#### 2.2.2. Nuclear Magnetic Resonance Analysis

The βCD–B complex was further confirmed by ^1^H NMR and ^11^B NMR using a Bruker AVANCE III spectrometer (Billerica, MA, USA) operated at 500 MHz. DMSO-d6 was used as the solvent and tetramethylsilane (TMS) was used as an internal reference for ^1^H NMR. The solutions were transferred into 5 mm NMR tubes to a total sample height of 4 cm. The probe temperature was maintained at 25 °C. Chemical shifts were expressed in parts per million downfield from the signal (0 ppm) of TMS.

#### 2.2.3. Differential Scanning Calorimetry (DSC) Analysis

DSC (SDT Q600, TA instruments, New Castle, DE, USA) was used to study the interactions between pMDI resin, the βCD-B complex and southern pine wood flour. Samples (10 ± 0.5 mg) of pMDI resin, wood, βCD–B and a physical mixture of any two or all three components at an equal weight ratio was weighed into aluminum pans and sealed with a pierced lid. The measurements were performed under a nitrogen atmosphere with a flow rate of 100 mL/min and all the samples were subjected to the same thermal history with the following protocol. First, the samples were heated from 26 to 120 °C at a heating rate of 10 °C/min and cooled to room temperature. This process was repeated two times to erase the thermal history and reduce variations in data interpretation. Second, the samples were heated from 26 to 250 °C at a heating rate of 10 °C/min to determine their thermal properties. Two replicate runs were performed for each sample.

### 2.3. Preparation of Oriented Strand Board (OSB)

**Figure 2:**
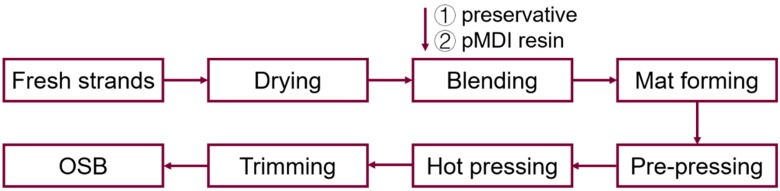
Flow chart of βCD–B-complex-treated Oriented Strand Board manufacturing.

Test panels were made under lab conditions (Figure 2). First, southern pine wood strands (Norbord Inc, Guntown, MS, USA) were dried to 6% moisture content and were mixed with 0%, 5% or 10% preservative (based on the oven-dried weight of strands), corresponding to boric acid equivalent (BAE) levels of 0%, 1.5% and 3.0%, respectively, in a rotary laboratory-type blender at a rotating speed of 20 rpm for 2 min. Subsequently, 0.04 g polymeric diphenylmethane diisocyanate (pMDI) resin (Huntsmen, Conroe, TX, USA) per g of wood was sprayed into the rotating drum using a spray gun with a nozzle size of 1.0 mm, followed by another 3 min of blending. The strands were transferred into a 254 × 254 mm mold randomly by hand. The formed mats were pressed at 176 ± 3 °C for 200 s to a target thickness of 11.7 mm. The panels were conditioned at 21 °C and 64% relative humidity (RH) before testing.

### 2.4. Internal Bond Strength and Fungal Resistance of OSB

Prior to examining the thickness-wise cohesion and fungal decay resistance of the panel, the vertical density profiles (VDPs) of the samples were measured using a QMS Density profiler (model QDP-01 X, Quintek Measurement Systems, Inc. Oak Ridge, TN, USA). Twenty-four internal bonding (IB) test samples (51 × 51 × 11.7 mm) of each treatment were weighed to a precision of 0.01 g. Three end-matched fungal decay test samples (14 × 14 × 11.7 mm) were cut from a 14 mm-wide strip next to each IB sample. The VDPs data were normalized using linear interpolation in accordance with the sample thickness, and the averaged VDPs of each treatment were reported. The samples with a squared residual (r^2^) greater than 0.56 were used for internal bonding tests.

The IB strength of the samples was examined as described in the ASTM D1037-12 standard [20]. Both wide faces of the samples were attached to the aluminum alloy holding blocks using a hot melt glue (Type 34614C, Applied Adhesives, MN, USA). A tensile loading perpendicular to sample surfaces was applied at a rate of 0.935 mm/min using a universal testing instrument (Instron 4204, Instron Corporation, Norwood, MA, USA). The IB strength was calculated using the following Equation

(1) $$\mathrm{IB}\;\mathrm{strength}\;\left(\mathrm{MPa}\right)=\frac{\mathrm{maximum}\;\mathrm{load}\;\left(N\right)}{\mathrm{length}\;\left(\mathrm{mm}\right)\times \mathrm{width}\;\left(\mathrm{mm}\right)}$$

Before exposure to fungi, the long-term performance of βCD–B-treated OSB samples was tested following the leaching procedures described in AWPA Standard E11-16 [21]. Briefly, half of the fungal decay test samples were placed into beakers and weighed down to prevent floating. A total of 240 mL of deionized water was added to each beaker, and the beakers were placed at room temperature on an orbital shaker rotating at a speed of 100 rpm. After 6, 24, 48 h, and thereafter at 48 h intervals, the leachate was removed from the beakers, and replaced with 100 mL fresh deionized water. The leaching process was performed for a total of 14 days.

The effect of the βCD–B complex on the decay resistance of the treated OSB samples was evaluated following AWPA Standard E10 [22]. Both unleached and leached specimens were sterilized by dipping the samples in 70% ethanol for 10 s and drying in a laminar flow cabinet, followed by the exposure of samples to one of two brown-rot fungi, *Gloeophyllum trabeum* (ATCC isolate 11539) and *Postia placenta* (ATCC 11538) for eight weeks. The mass loss was calculated according to the following Equation

(2) $$\mathrm{Mass}\;\mathrm{loss}\;\left(\%\right)=\frac{\left(m-m_{expo.}\right)}{m}\times 100\%$$

where m and m*_exp._* are the oven-dried mass of untreated or treated OSB cubes before and after exposure to fungi, respectively.

### 2.5. Statistical Analysis

Statistical analysis was performed using Statistical Analysis System software (SAS version 9.4, SAS Institute, Cary, NC). The data were compared using a one-way analysis of variance (ANOVA) followed by post-hoc Fisher’s Least Significant Difference (*p* < 0.05) to identify the effects of preservative levels on the IB, average densities and mass loss of the panels after exposure to fungi.

## 3. Results and Discussion

### 3.1. Formation of βCD–B Complex

**Figure 3:**
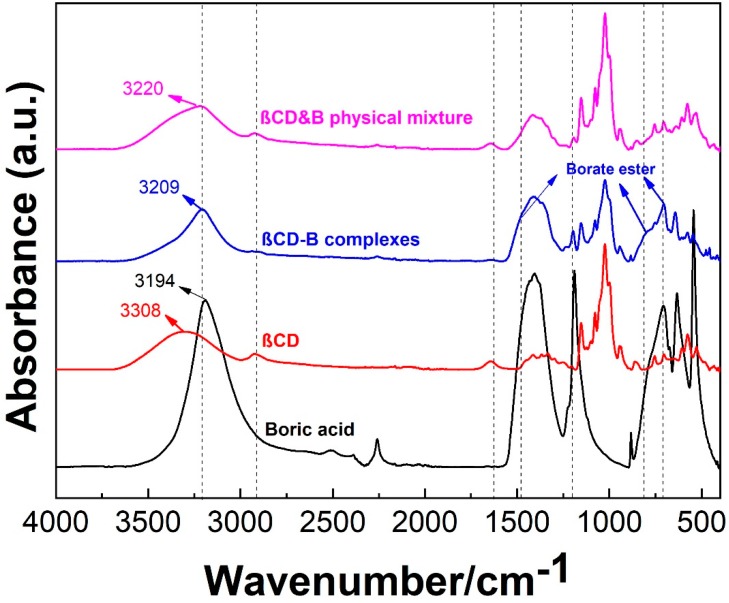
Infrared spectra of boric acid, βCD, βCD–B complex and their physical mixture.

The formation of the βCD–B complex was evident by changes in the IR spectra of the complex, as compared to the spectra of the individual components and physical mixture (Figure 3). The FTIR spectrum of boric acid exhibits absorption bands at 3193 and 1409 cm^−1^ due to the stretching of O–H and asymmetric stretching of the B–O bond, respectively [23]. In the spectrum of the βCD, a sharp absorption band at 3308 cm^−1^ and an intense peak at 1078 cm^−1^ due to stretching of the O–H and C–O bond, respectively, were seen. The 2924 cm^−1^ peak corresponds to C–H stretching while the 1367 cm^−1^ peak can be attributed to scissoring of the C–H group [24]. Upon reaction with boric acid, the O–H band in the complex shifted to a lower frequency (~3209 cm^−1^) and became narrower, as compared to those of the physical mixture (~3220 cm^−1^) and pure βCD (~3308 cm^−1^). Also, the intensity of O–H stretching at 1645 cm^−1^ was greatly reduced in the βCD–B complex. These observations suggest that a fraction of the O–H groups in βCD are substituted by boric acid, as shown in Figure 3. Moreover, the C–H stretching (~2924 cm^−1^) in the βCD–B complex exhibited a lower intensity and shifted to a lower frequency, which possibly resulted from an altered environment around these bonds upon complexation. These results were also supported by the appearance of new borate ester B (OR)_3_ peaks at 1473, 805, 728 cm^−1^. Therefore, the formation of the βCD–B complex was indicated by the changes in OH groups and the formation of new peaks attributed to borate ester [25].

**Figure 4:**
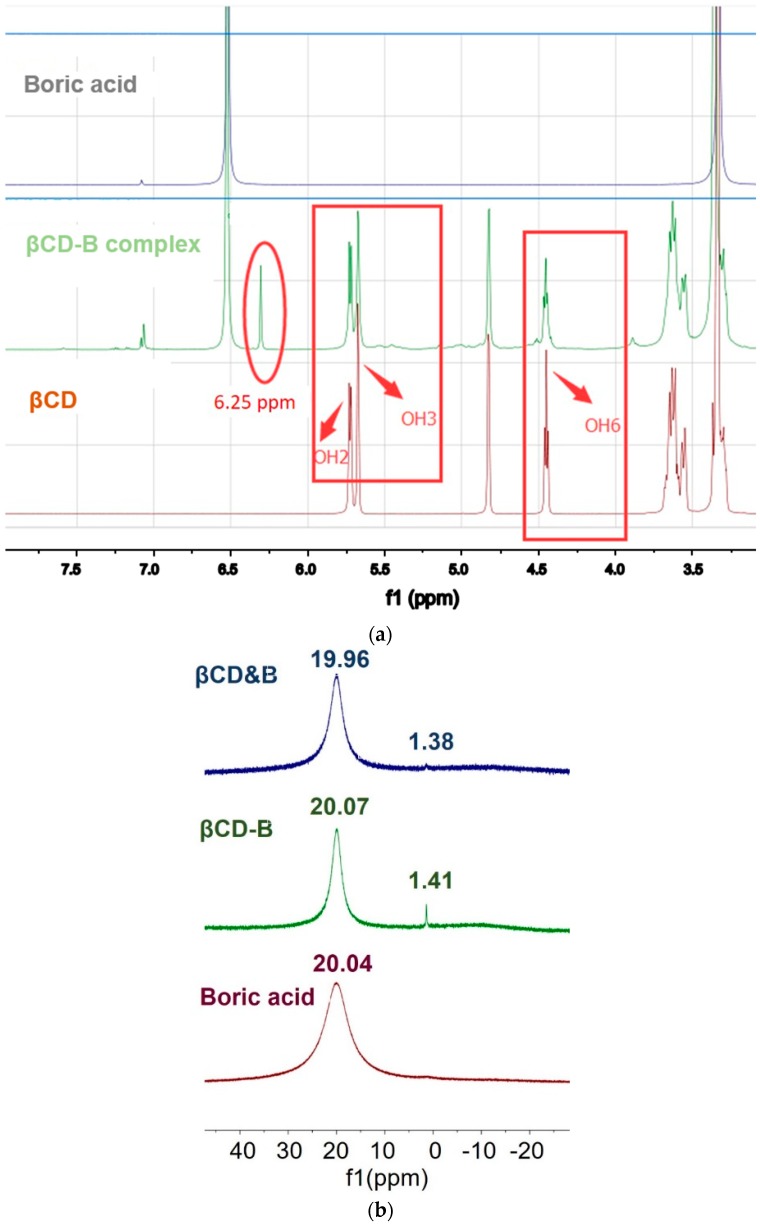
(**a**) ^1^H Nuclear magnetic resonance (NMR) of boric acid, βCD–B complex and βCD in DMSO-D6 (TMS as internal references) and (**b**) B-11 NMR of boric acid, βCD-B complex and βCD&B physical mixture in DMSO-D6 (normalized).

The ^1^H NMR results of boric acid, βCD, and βCD–B complex are shown in Figure 4a. A new peak at 6.25 ppm was observed in the ^1^H NMR spectrum of βCD–B, as compared to that of the neat βCD. Also, the decreased intensities in the peaks of H-3 and H-6 relative to the peak of H-2 in βCD–B suggest that borate esters are replacing the OH groups in βCD. According to the NMR results, the OH groups in boric acid may either attach at the OH sites corresponding to the H-2, H-3 or H-6 peaks in cyclodextrins [26].

The complexation between βCD and boric acid was also confirmed by ^11^B NMR. Figure 4b shows the normalized ^11^B NMR spectra of boric acid, βCD–B complex and the physical mixtures. Only one peak (at around 20 ppm) was observed in the ^11^B NMR spectra of boric acid. Conversely, there was a new peak at 1.41 ppm in the ^11^B NMR spectrum of the βCD–B complex, which was assigned to the B–O–C bond containing residual-OH groups from boric acid [27]. A small peak at 1.38 ppm was also observed in the βCD&B physical mixture due to the tendency of poly-condensation between boric acid and polyols and the prevention of hydrolysis by using DMSO-d6 as a solvent. 

### 3.2. Curing Behavior of Polymeric Methylene Diphenyl Diisocyanate (pMDI), Wood, βCD-B Complex and Their Mixtures

**Figure 5:**
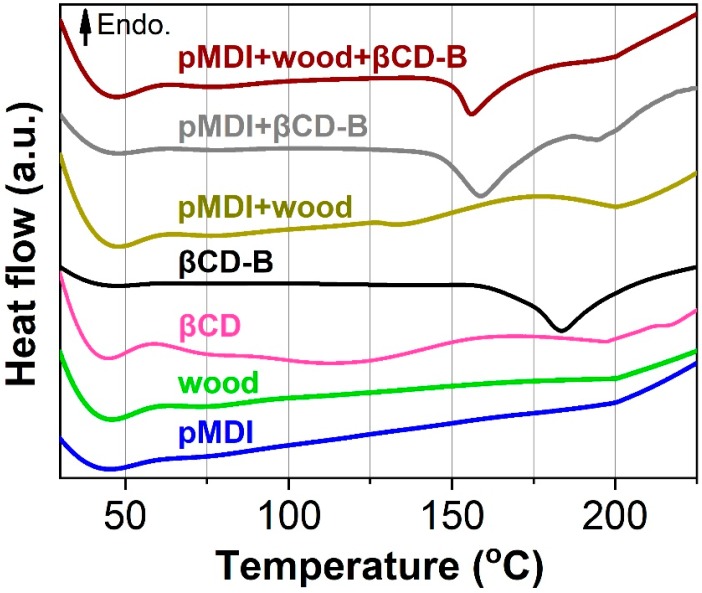
Differential scanning caliometry (DSC) curves of pMDI resin, βCD–B complex, wood and their physical mixture.

The DSC curves of pMDI resin, wood, βCD, βCD–B complex, and the physical mixture of any two, or all three, of the components at equal weights are shown in Figure 5. An exothermic peak was observed in the βCD–B complex (Figure 5, black curve; compare to the pink curve below). This peak could be attributed to the decomposition of the βCD–B complex, and similar results with a higher decomposition temperature have been reported when starch was esterified by boric acid [28]. Moreover, this peak centered at 183 °C in the βCD–B complex shifted to the lower temperature of 158 °C in the presence of the pMDI resin and shifted slightly further to 156 °C when blended with wood. This fact could be related to the high reactivity rate of decomposed boron towards isocyanate-containing resins [29]. The decreased exothermic temperature in the mixture of resin, wood and preservatives indicates that the addition of βCD-B complex possibly lowered the temperature required to initiate the curing reaction of resin, as boron compounds have been used to catalyze the polymerization in isocyanate-based resin systems [11,30,31]

### 3.3. Vertical Density Profile (VDP) and Internal Bonding of the Panel

**Figure 6:**
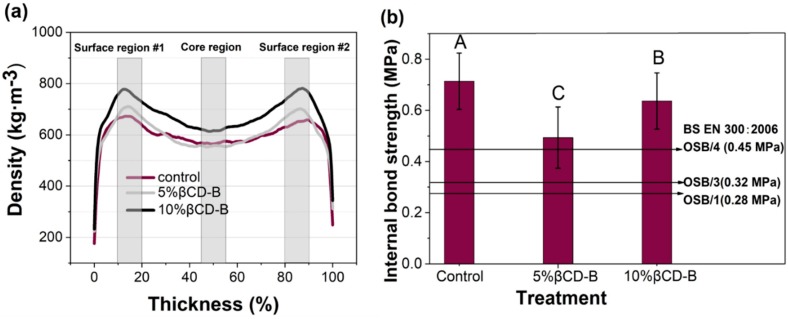
(**a**) Average vertical density profiles normalized based on the thickness of each panel, and (**b**) internal bond strength of oriented strand boards (OSB) at 0%, 5% and 10%) βCD–B treatment.

The average VDPs, normalized based on the thicknesses of the IB test specimens of each panel, are presented in Figure 6a, which are characterized by high-density near the panel surfaces and low-density at the panel cores. VDPs are determined by hot press manufacturing parameters such as the moisture distribution in the mat, pressing schedules, and platen temperatures [32]. The average densities of the panels near the surface regions (10%–20% and 80%–90% of the normalized thicknesses) and at core regions (45%–55% of the normalized thicknesses) are shown in Table 1. The average densities of the surface regions were significantly higher than the core regions for all three treatments. The average densities of the surface regions #1 and #2 at 5% preservative treatment increased by 7% and 9%, respectively, while the densities of the core regions were not significantly affected as compared to those of the control. The addition of 10% preservative increased the densities of the surface regions #1 and #2 by 16% and 19%, respectively, while it increased the core density by 8% compared to the control. These results indicate that the preservative addition densified the surface layers more than the core layers and affected compaction throughout the thicknesses of the treated panels. As discussed in the previous section, the boron-based preservative particles possibly lowered the curing temperature of the pMDI resin and accelerated densification near the panel surfaces. Such densification then would affect the mass and heat movements toward the panel cores and decrease their compactness ratios from the control [33,34].

The average IB strength values of the panel as a function of the preservative level are presented in Figure 6b. The addition of preservatives significantly decreased the cohesion strength of the panels, mainly by reducing the compaction ratios of the panel cores, as discussed earlier. Additionally, due to the low water solubility of the βCD–B complex, most of the compounds would sit in a powder form on the surface of the wood during hot pressing, resulting in a lager surface area of solids within the mat and a lower bonding efficiency of the resin [11]. Meanwhile, a significant increase in IB strength was observed when the preservative loading was increased from 5% to 10%. The increased amount of boric acid in the βCD–B complex may have acted as a crosslinking agent that improved the bonding strength of the pMDI resin in the presence of steam [29,35]. Further investigation on the interactions between βCD–B, pMDI, and wood in the presence of water is needed. Although the addition of the βCD–B complex overall significantly reduced the internal bonding of the panel, their IB was above the BS EN 300 standard for load-bearing boards for use in humid conditions (Type OSB/3) [36].

### 3.4. Decay Resistance of βCD–B Complex Against Brown-Rot Fungi

**Figure 7:**
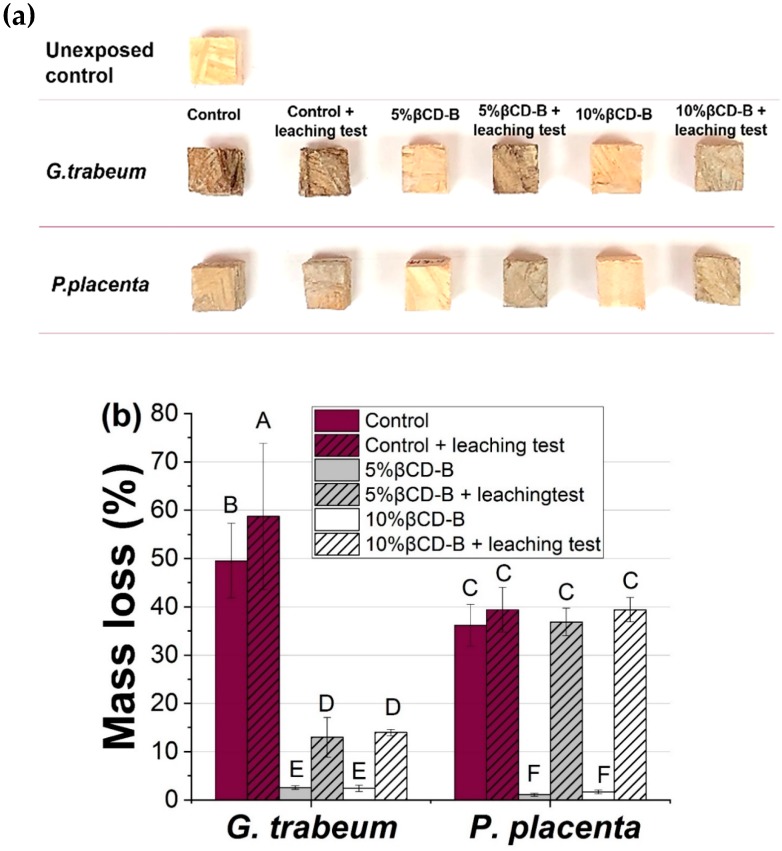
(**a**) Photograph and (**b**) mass loss of OSB treated with 0%, 5% and 10% βCD–B after eight week exposure to G. trabeum and P. placenta. Note: Means designated by the same letter are not significantly different (LSD, *p* < 0.05).

The fungal resistance of southern pine OSB panels treated with βCD–B at levels of 0%, 5% and 10% was tested against *G. trabeum* and *P. placenta* for an incubation time of eight weeks. Figure 7a shows the unexposed control and decayed OSB samples. The unleached, treated OSB cubes showed limited decay as compared to the extensively colonized control and treated, leached samples. The mass loss of OSB samples is shown in Figure 7b. The average percent mass loss caused by *G. trabeum* in the untreated control group (both unleached and leached) showed a mass loss greater than 45%, indicating a high activity of the fungus used for testing. In comparison, the mass loss of unleached OSB treated with 5% βCD–B complex was significantly decreased to approximately 3% (*p* < 0.05), which is not significantly different from the mass loss of OSB samples containing 10% βCD–B complex, as both treatment levels were above the preservative threshold of 1% (BAE) [37]. This observation also indicates that the bioavailability of boron in the βCD–B complex was not affected by encapsulation. In the case of leached samples, the mass losses of 5% and 10% βCD–B complex treatments were not significantly different (~15%, *p* < 0.05), but still significantly lower than the mass loss of control groups (50%–60%, *p* < 0.05). These results indicate that the βCD–B complex or boric acid are leached from the samples, resulting in reduced resistance against the fungus.

Unleached, treated samples exposed to *P. placenta* also showed low mass losses, indicating that *P. placenta* and *G. trabeum* might have a similar BAE threshold, as reported by Freitag and Morrell [38]. However, the leached samples treated with both preservative levels (at 5% and 10% βCD–B) showed a significantly higher mass loss than those exposed *to G. trabeum* (*p* < 0.05). In this study, the mass loss differences in the leached samples in these two fungi could result from the variations in the panels selected for the soil block test, as the decay test specimens for *G. trabeum* and *P. placenta* were selected from different panels. Another reason could be related to more delamination of specimens during *P. placenta* exposure under warm and humid conditions, which could lead to high leaching rates [39].

**Figure 8:**
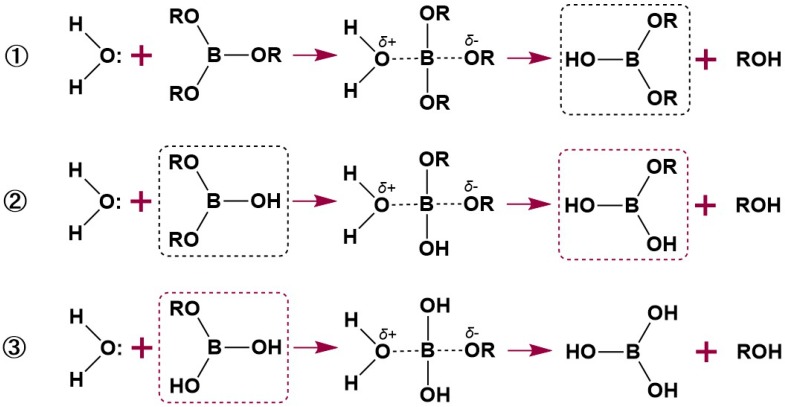
The hydrolysis mechanism of borate esters. Adapted from Steinberg and Hunter, 1957 [41].

The leaching of boric acid in this study, indicated by the increased mass losses of leached samples, likely results from the hydrolysis of boric acid from βCD–B. In this study, βCD–B was obtained by the esterification reaction between boric acid and cyclodextrin. It has been reported that the greater the bulk of the R group in the borate esters (Figure 8), the slower the hydrolysis rate [40,41]. However, the intrinsic low hydrolysis stability of boric acid esters remains [41] because of the empty *p*-orbital in boron [42]. In other words, three of the hybridized sp2 orbitals form covalent bonds with other atoms, while one unhybridized *p*-orbital remains unoccupied. The vacant *p*-orbital imparts the compounds with Lewis acid character and tends to accept unshared electrons from donor species, e.g., water molecules [25]. Therefore, due to the tendency of boron to be attacked by water and the harsh leaching conditions employed in this study, boric acid might be released from the βCD–B complex, consequently compromising the durability of the fungal exposed samples. Bioavailable borate is necessary for wood protection, and permanently immobilized borates would reduce the biological activity of borate complexes [43,44]. Therefore, it might be beneficial to design borate esters with a controlled hydrolysis rate and evaluate their biological performance over time.

## 4. Conclusions

The difference between the mass loss of control group and βCD–B-complex-treated OSB indicates the suitability of βCD–B as a potential wood preservative for wood composites’ protection. Boric acid can be partially fixed by βCD and the bioavailability of the βCD–B complex is not affected by encapsulation. The internal bond strength of the βCD–B-preservative-treated panel meets the minimum requirement for the European standard for load-bearing boards for use in humid conditions. However, the βCD–B complex is prone to hydrolysis, thus the constructed OSB could only be used where there is a minimum leaching hazard. Future research will continue to focus on improving the leaching performance of βCD–B-complex-treated OSB. The mold resistance and mechanical properties of the treated panels made, implementing the optimized manufacturing parameters, will also be investigated.

## Figures and Tables

**Table 1:** Average density (± standard error) at different regions in OSB panels.

Preservatives Level (%)	Average Density ± Standard Error (kg/m^3^)		
	Surface Region #1	Core Region	Surface Region #2
0 *	665 ± 24 (C)	562 ± 28 (A)	652 ± 30 (C)
5	712 ± 33 (D)	568 ± 24 (A)	711 ± 30 (D)
10	770 ± 36 (E)	606 ± 26 (B)	774 ± 40 (E)

Note: Means followed by the same letter are not significantly different (LSD, *p* < 0.05). * Data from a submitted manuscript [16].

## References

[1] Gardner D.J., Tascioglu C., Wålinder M.E.P. (2003). Wood Composite Protection. Wood Deterioration and Preservation.

[2] Goodnight C.J. (1942). Toxicity of Sodium Pentachlorophenate and Pentachlorophenol to Fish. Ind. Eng. Chem..

[3] Hall A.H. (2002). Chronic Arsenic Poisoning. Toxicol. Lett..

[4] Williams L.H. (1996). Borate Wood-Protection Compounds: A Review of Research and Commercial Use. APT Bull..

[5] Freeman M.H., Mcintyre C.R., Associates M. A Critical and Comprehensive Review of Boron in Wood Preservation. Proceedings of the American Wood Protection Association.

[6] Cabrera Y., Morrell J.J. (2009). Effect of Wood Moisture Content and Rod Dosage on Boron or Fluoride Movement through Douglas-Fir Heartwood. For. Prod. J..

[7] Caldeira F. (2010). Boron in Wood Preservation: A Review in Its Physico-Chemical Aspects. Silva Lusit..

[8] Obanda D.N., Shupe T.F., Barnes H.M. (2008). Reducing Leaching of Boron-Based Wood Preservatives—A Review of Research. Bioresour. Technol..

[9] Tsunoda K. (2001). Preservative Properties of Vapor-Boron-Treated Wood and Wood-Based Composites. J. Wood Sci..

[10] Manning M.J. (2002). Wood Protection Processes for Engineered Wood Products. Proceedings of the Enhancing the Durability of Lumber and Engineered Wood Products, FPS Symposium Proceedings.

[11] Laks P.E. The Effects of Sodium Octaborate Tetrahydrate and Zinc Borate on the Properties of Isocyanate-Bonded Waferboard. Proceedings of the Adhesives and Bonded Wood Products Symposium.

[12] Szejtli J. (1988). Cyclodextrin Technology.

[13] Bhat S., Chandrasekaran S. (1996). Oxygenation of Alkenes with T-BuOOH Catalysed by β-Cyclodextrin Borate. Tetrahedron Lett..

[14] Baur R., Macholdt H.-T. (2000). Cyclooligosaccharide-Boron Complex. US Patent.

[15] Cai L., Lim H., Nicholas D.D., Kim Y. (2020). Bio-Based Preservative Using Methyl-β-Cyclodextrin-Essential Oil Complexes for Wood Protection. Int. J. Biol. Macromol..

[16] Cai L., Lim H., Kim Y., Jeremic D. (2019). β-Cyclodextrin-Allyl Isothiocyanate Complex as a Natural Preservative for Wood Products.

[17] Cai L., Lim H., Kim Y., Jeremic D. (2019). β-Cyclodextrin-Allyl Isothiocyanate Complex as a Natural Preservative for Strand-Based Wood Composites.

[18] Mohamed M.H., Wilson L.D., Headley J.V. (2011). Design and Characterization of Novel β-Cyclodextrin Based Copolymer Materials. Carbohydr. Res..

[19] Kawano S., Kida T., Miyawaki K., Fukuda Y., Kato E., Nakano T., Akashi M. (2015). Adsorption Capability of Urethane-Crosslinked Heptakis (2, 6-Di-O-Methyl)-β-Cyclodextrin Polymers toward Polychlorobiphenyls in Nonpolar Organic Media. Polym. J..

[20] ASTM International (2012). ASTM D1037-12 Standard Test Methods for Evaluating Properties of Wood-Base Fiber and Particle Panel Materials.

[21] AWPA (2016). E11-16: Standard Method for Accelerated Evaluation of Preservative Leaching. AWPA Book of Standard.

[22] AWPA (2016). E10-16: Laboratory Method for Evaluating the Decay Resistance of Wood-Based Materials against Pure Basidiomycete Cultures: Soil/Block Test. AWPA Book of Standard.

[23] Jun L., Shuping X., Shiyang G. (1995). FT-IR and Raman Spectroscopic Study of Hydrated Borates. Spectrochim. Acta Part A Mol. Biomol. Spectrosc..

[24] Kemelbekov U., Luo Y., Orynbekova Z., Rustembekov Z., Haag R., Saenger W., Praliyev K. (2011). IR, UV and NMR Studies of β-Cyclodextrin Inclusion Complexes of Kazcaine and Prosidol Bases. J. Incl. Phenom. Macrocycl. Chem..

[25] Li X.N., Zheng G., Yang H.X. (2010). Study on Synthesis and Properties of a Novel Borate Ester Surfactant. Advanced Materials Research.

[26] Schneider H.-J., Hacket F., Rüdiger V., Ikeda H. (1998). NMR Studies of Cyclodextrins and Cyclodextrin Complexes. Chem. Rev..

[27] Ahmadi Y., Siddiqui M.T., Haq Q.M.R., Ahmad S. (2018). Synthesis and Characterization of Surface-Active Antimicrobial Hyperbranched Polyurethane Coatings Based on Oleo-Ethers of Boric Acid. Arab. J. Chem..

[28] Staroszczyk H. (2009). Microwave-Assisted Boration of Potato Starch. Polimery.

[29] Aries R.S. (1960). Process and Product of Reacting Boric Acid with Isocyanates. US Patent.

[30] Ashida K. (1986). Boronium Complexes as Polymer Catalysts. UK Patent.

[31] Lucey M.F. (1981). Electronic Component with Radiation-Hardenable Coating. US Patent.

[32] Wang S., Winistorferl P.M. (2000). Density Formation Under Dynamic Conditions. Wood Fiber Sci..

[33] Wong E.D. (1998). Effects of Mat Moisture Content and Press Closing Speed on the Formation of Density Profile and Properties of Particleboard. J. Wood Sci..

[34] Wong E.D., Zhang M., Wang Q., Kawai S. (1999). Formation of the Density Profile and Its Effects on the Properties of Particleboard. Wood Sci. Technol..

[35] Leventis N., Sotiriou-Leventis C., Saeed A.M., Donthula S., Majedi Far H., Rewatkar P.M., Kaiser H., Robertson J.D., Lu H., Churu G. (2015). Nanoporous Polyurea from a Triisocyanate and Boric Acid: A Paradigm of a General Reaction Pathway for Isocyanates and Mineral Acids. Chem. Mater..

[36] BSI (2006). BS EN 300:2006. Oriented Strand Boards (OSB). Definitions, Classification and Specifications.

[37] Xu X., Lee S., Wu Y., Wu Q. (2013). Borate-Treated Strand Board from Southern Wood Species: Resistance against Decay and Mold Fungi. BioResources.

[38] Freitag C., Morrell J.J. (2005). Development of Threshold Values for Boron and Fluoride in Non-Soil Contact Applications. For. Prod. J..

[39] Evans P.D., Lube V., Averdunk H., Limaye A., Turner M., Kingston A., Senden T.J. (2015). Visualizing the Microdistribution of Zinc Borate in Oriented Strand Board Using X-Ray Microcomputed Tomography and SEM-EDX. J. Compos..

[40] Hall D.G. (2006). Structure, Properties, and Preparation of Boronic Acid Derivatives. Overview of Their Reactions and Applications. Boronic Acids Prep. Appl. Org. Synth. Med..

[41] Steinberg H., Hunter D.L. (1957). Preparation and Rate of Hydrolysis of Boric Acid Esters. Ind. Eng. Chem..

[42] Matsumi N., Naka K., Chujo Y. (1998). Extension of π-Conjugation Length via the Vacant p-Orbital of the Boron Atom. Synthesis of Novel Electron Deficient π-Conjugated Systems by Hydroboration Polymerization and Their Blue Light Emission. J. Am. Chem. Soc..

[43] Efhamisisi D., Thevenon M., Hamzeh Y., Pizzi A., Karimi A., Pourtahmasi K. (2017). Tannin-Boron Complex as a Preservative for 3-Ply Beech Plywoods Designed for Humid Conditions. Holzforschung.

[44] Tondi G., Wieland S., Lemenager N., Petutschnigg A., Pizzi A., Thevenon M.F. (2012). Efficacy of Tannin in Fixing Boron in Wood: Fungal and Termite Resistance. BioResources.

